# Supplementary material for: Mutations in PCYT2 disrupt etherlipid biosynthesis and cause a complex hereditary spastic paraplegia
Source: Brain. 2019 Oct 22;142(11):3382–97. doi: 10.1093/brain/awz291 (PMC6821184; doi:10.1093/brain/awz291)
Supplement: awz291_Supplementary_Data [file awz291_supplementary_data.zip › awz291-Suppl_data/Supplementary_Data4.zip!/Supplementary information 4 - plasma lipidomics.zip!/Plasma lipidomics/Control vs Patient/Statistical plots and data/PA species/Boxplots.pdf]

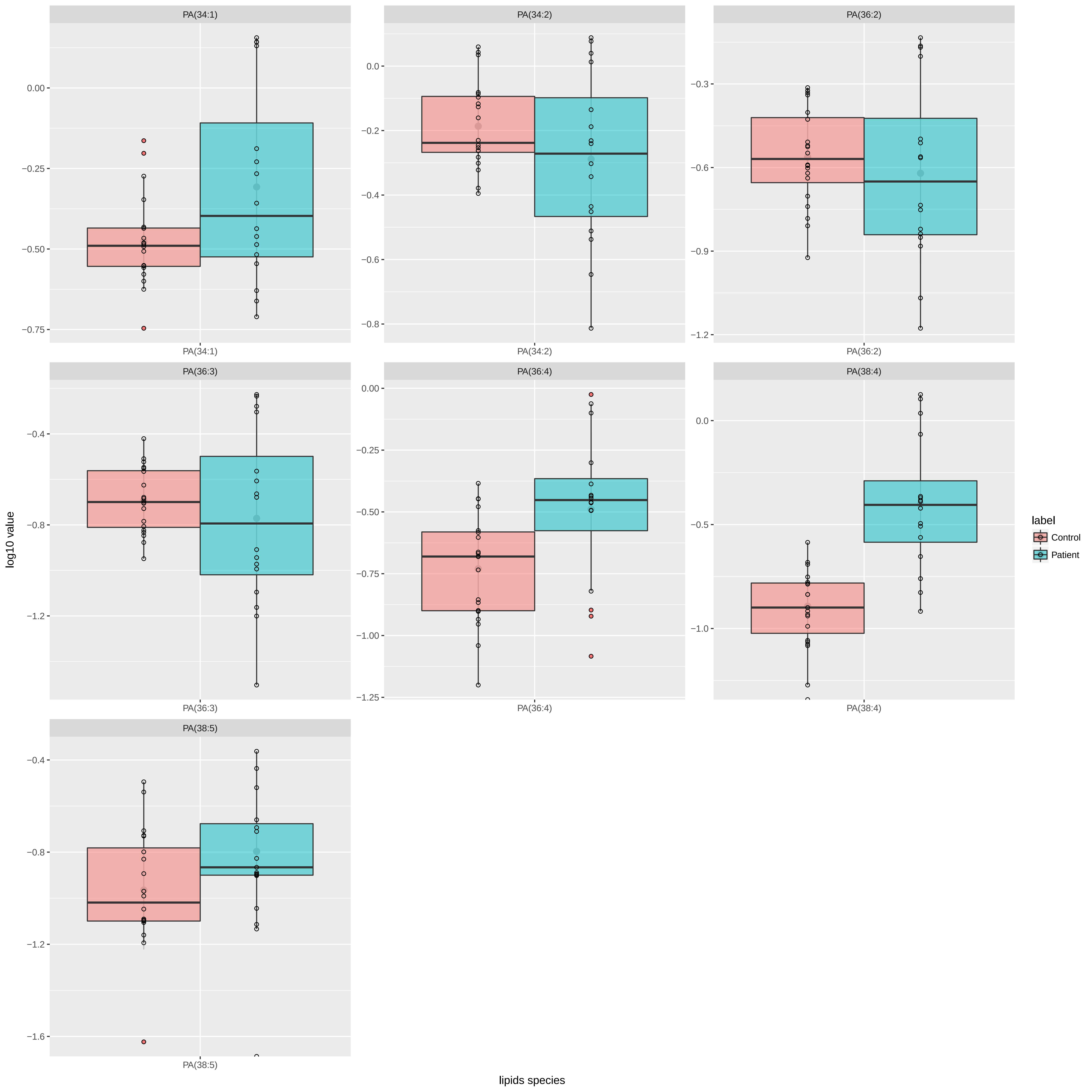
PA(34:1)
PA(34:2)
PA(36:2)
PA(36:3)
PA(36:4)
PA(38:4)
PA(38:5)
0.00
−0.25
−0.50
−0.75
0.0
−0.2
−0.4
−0.6
−0.8
−0.3
−0.6
−0.9
−1.2
−0.4
−0.8
−1.2
−1.25
−1.00
−0.5
−1.0
−1.6
log10 value
lipids species
label
Control
Patient
